# Systematic review and meta-analysis of biomarkers of sarcopenia and sarcopenic obesity

**DOI:** 10.1097/MD.0000000000050799

**Published:** 2026-09-25

**Authors:** Yaxian Yang, Chunyu Wang, Ruiyuan Xu, Wang Xuan, Xiaoai Chen

**Affiliations:** a Department of Endocrinology, the 940th Hospital Joint Logistics Support Forces of PLA, Lanzhou, Gansu, China.

**Keywords:** biomarkers, hematologic markers, inflammation, sarcopenia, sarcopenic obesity

## Abstract

**Objective:**

Sarcopenia and sarcopenic obesity (SO) are geriatric syndromes characterized by reduced skeletal muscle mass and impaired muscle strength, whose specific biomarkers and underlying mechanisms remain unclear. This study aimed to conduct a meta-analysis and literature review on the associations between biomarkers and sarcopenia/SO by collecting relevant publications over the past 15 years. We intended to further identify the shared and differential biomarkers of these disorders, thereby revealing their distinct pathophysiological processes and providing a basis for precise diagnosis and targeted intervention.

**Methods:**

A comprehensive literature search was performed in PubMed, Embase databases for all publications from December 2010 to December 2025. We included English articles describing the associations between serum biomarkers and sarcopenia/SO, including cross-sectional studies, prospective cohort studies, and retrospective case studies, with no restrictions on race, population, or sample size. Literature screening was conducted in accordance with predefined inclusion and exclusion criteria. Relevant study data were extracted, and the quality of included studies was assessed. The mean difference and 95% confidence interval were used to calculate the pooled effect size.

**Results:**

A total of 43 English articles investigating the associations between biomarkers and sarcopenia/SO were finally included. Compared with non-sarcopenic patients, sarcopenic patients had elevated serum levels of C-reactive protein (CRP), interleukins, tumor necrosis factor, cystatin C, blood urea nitrogen, and high-density lipoprotein cholesterol (HDL-C). In contrast, higher serum levels of vitamin D, alanine transaminase, albumin, hemoglobin, TG, uric acid, blood glucose, and fasting insulin were associated with a lower incidence of sarcopenia. No significant differences were observed in WBC count, platelet count, aspartate transaminase, alkaline phosphatase, bilirubin, total cholesterol (TC), creatinine, erythrocyte sedimentation rate, low-density lipoprotein cholesterol (LDL-C), or thyroid-stimulating hormone levels between sarcopenic and non-sarcopenic patients. Compared with healthy individuals, SO patients had higher serum levels of CRP, LDL-C, TC, TG, WBC, creatinine, uric acid, fasting blood glucose, and fasting insulin, while higher levels of HDL-C and vitamin D were associated with a lower risk of SO. SO patients had lower TG levels than patients with simple obesity, with no significant differences in CRP, HDL-C, LDL-C, TC, vitamin D, blood glucose, or interleukin-6 levels between the 2 groups.

## 1. Introduction

Aging is a complex process characterized by progressive decline in organ function, disruption of homeostasis, reduction in physiological reserve, and alterations in body composition.^[1]^ The aging process is accompanied by various changes in the human body, among which skeletal muscle mass begins to decline gradually from the age of 30 and accelerates after 65 years old, along with a decrease in muscle strength.^[2]^ Sarcopenia refers to the age-related loss of muscle mass and function. With the accelerated global population aging, the incidence of sarcopenia and obesity in the elderly is rising year by year, sarcopenic obesity (SO) is a type of obesity marked by the decline in muscle mass, muscle strength, and physical performance.^[3]^ The combination of sarcopenia and obesity, known as SO, has become a major public health issue threatening the health of the elderly and a research hotspot in geriatrics due to its higher risk of adverse clinical outcomes. According to the latest epidemiological surveys, the global prevalence of SO in the elderly is approximately 11%, and it may be even higher in regions with high rates of malnutrition and chronic diseases. SO is closely associated with various diseases such as cardiovascular disease, diabetes mellitus, chronic respiratory disease, and nonalcoholic fatty liver disease, significantly increasing the risk of disability, mortality, and medical burden in the elderly.^[4]^ At present, the pathogenesis of SO has not been fully elucidated, and its clinical phenotype is complex, involving multiple pathological processes such as inflammatory response, metabolic disorder, mitochondrial dysfunction, and oxidative stress. Studies have shown that SO patients have a worse prognosis than those with simple obesity or sarcopenia, with higher risks of cardiovascular events, respiratory complications, liver disease, physical dysfunction, and death.^[5]^ However, early screening and diagnosis of SO still face great challenges due to the lack of specific biomarkers and a unified clinical evaluation system, leading to delayed intervention and impaired disease management efficacy.^[6]^

In recent years, scholars at home and abroad have carried out extensive research on the clinical characteristics and biomarkers of sarcopenia and SO. However, discrepancies in population characteristics and detection methods across different studies have led to the absence of a unified conclusion on the correlation patterns of some biomarkers. Moreover, the number of relevant studies is limited, and there is a lack of meta-analyses based on original research. This study aims to investigate the association between various biomarkers (including inflammatory factors, hormone-related markers, oxidative stress response markers and circulating mediators) and the risk of sarcopenia and SO by means of meta-analysis, so as to provide evidence for the diagnosis, treatment and prognosis of sarcopenia.

## 2. Materials and methods

### 2.1. Literature search

We searched all English articles published from December 2010 to December 2025 on the associations between sarcopenia, SO and relevant biomarkers in 2 major English databases, PubMed and EMBASE, and additionally traced the references of the retrieved articles. We performed this systematic review and meta-analysis in accordance with the Preferred Reporting Items for Systematic Reviews and Meta-Analyses Statement.^[7]^ Based on the PICOS principle, a search strategy combining Mesh terms and free words was adopted, and the retrieval was conducted using the following search formula: (“Sarcopenia”[Mesh] OR “SO”[Mesh]) AND (“Biomarkers inflammation”[Mesh] OR “Hematologic markers”[Mesh]).

### 2.2. Inclusion and exclusion criteria

**Inclusion criteria**: Human-based studies with a diagnosis of muscle atrophy accompanied by muscle weakness and/or physical dysfunction^[8,9]^; Participants aged ≥50 years; Studies published in Chinese or English; Availability of mean and standard deviation (SD) data of biomarker levels;

**Exclusion criteria**: Studies without reporting the diagnostic criteria for sarcopenia or defining sarcopenia only by skeletal muscle mass, as well as animal experiments, reviews, meta-analyses, conference abstracts, comments. Duplicate publications, studies with incomplete data and poor quality were also excluded.

### 2.3. Data extraction

Two researchers independently conducted the literature search and determined whether the finally included articles met the above inclusion criteria. In case of inconsistent opinions, consensus was reached through mutual consultation. The basic characteristics of the included studies were extracted, including the following information: first author’s name, publication year, country of the study population, study type, sample size, number of participants in the experimental and control groups, and the mean and SD of relevant serum biomarkers in the 2 groups. If the mean and SD were not provided, the median and interquartile range of relevant indicators were extracted, and corresponding variable adjustments were made.^[10]^

### 2.4. Quality assessment

The Cochrane Collaboration tool^[11]^ was used to evaluate the included literatures, including random sequence generation, allocation concealment, blinding, completeness of outcome data, selective reporting, and other sources of bias. Supplementary Table S1, Supplemental Digital Content 1 shows the risk of bias of included literatures.

### 2.5. Statistical analysis

In this study, comparisons were performed between the sarcopenia/SO groups and the control group (healthy individuals), as well as between patients with SO and those with simple obesity. Subgroup analysis were conducted according to the countries where the study populations were recruited, which were stratified into the Asian region and European or American region. The total number of participants in each experimental and control group was extracted, followed by the extraction of the mean and SD of relevant serum biomarkers. For studies with original data presented as median and interquartile range, the mean and SD of the corresponding serum biomarkers were calculated using the statistical method described by Chen et al.^[10]^ mean difference and 95% confidence interval (CI) were then calculated based on the mean and SD of serum biomarkers in each group. When the pooled effect size was 0 or its 95% CI included 0, the diamond representing the pooled effect size in the forest plot intersected the line of no effect, indicating no statistically significant difference in the relevant outcome indicators between the experimental and control groups. When the effect size was >0 and the lower limit of its 95% CI was >0, the diamond was located on the right side of the line of no effect, indicating that the level of the relevant serum biomarker in the experimental group was higher than that in the control group. When the effect size was <0 and the upper limit of its 95% CI was <0, the diamond was located on the left side of the line of no effect, indicating that the level of the serum biomarker in the control group was higher than that in the experimental group. Forest plots were generated using Stata 12.0 statistical software. Heterogeneity was tested using the Cochran *Q* test and *I*^2^ statistic. A fixed-effects model was used if *I*^2^ <50%; otherwise, a random-effects model was applied. *P* value < .05 was considered statistically significant for the *Q* test, *I*^2^ statistic, and subgroup analysis. Sensitivity analysis was performed to evaluate the robustness of the pooled effect sizes. We adopted the leave-one-out approach, in which each individual study was sequentially excluded one at a time, and the pooled standardized mean difference (SMD) with corresponding 95% CIs was recalculated after each exclusion. If the pooled estimate did not change substantially after omitting a single study, the overall result was considered robust. All sensitivity analyses were carried out using Stata12.0 software.

## 3. Results

### 3.1. Search results

A total of 123 articles related to sarcopenia or SO were retrieved from PubMed and Embase databases, including 56 from PubMed and 67 from Embase. 13 duplicate articles were excluded. After reading the titles, abstracts, and full texts, 43 studies^[12–54]^ were finally included. The details of the screening process are shown in Figure 1.

**Figure 1.**
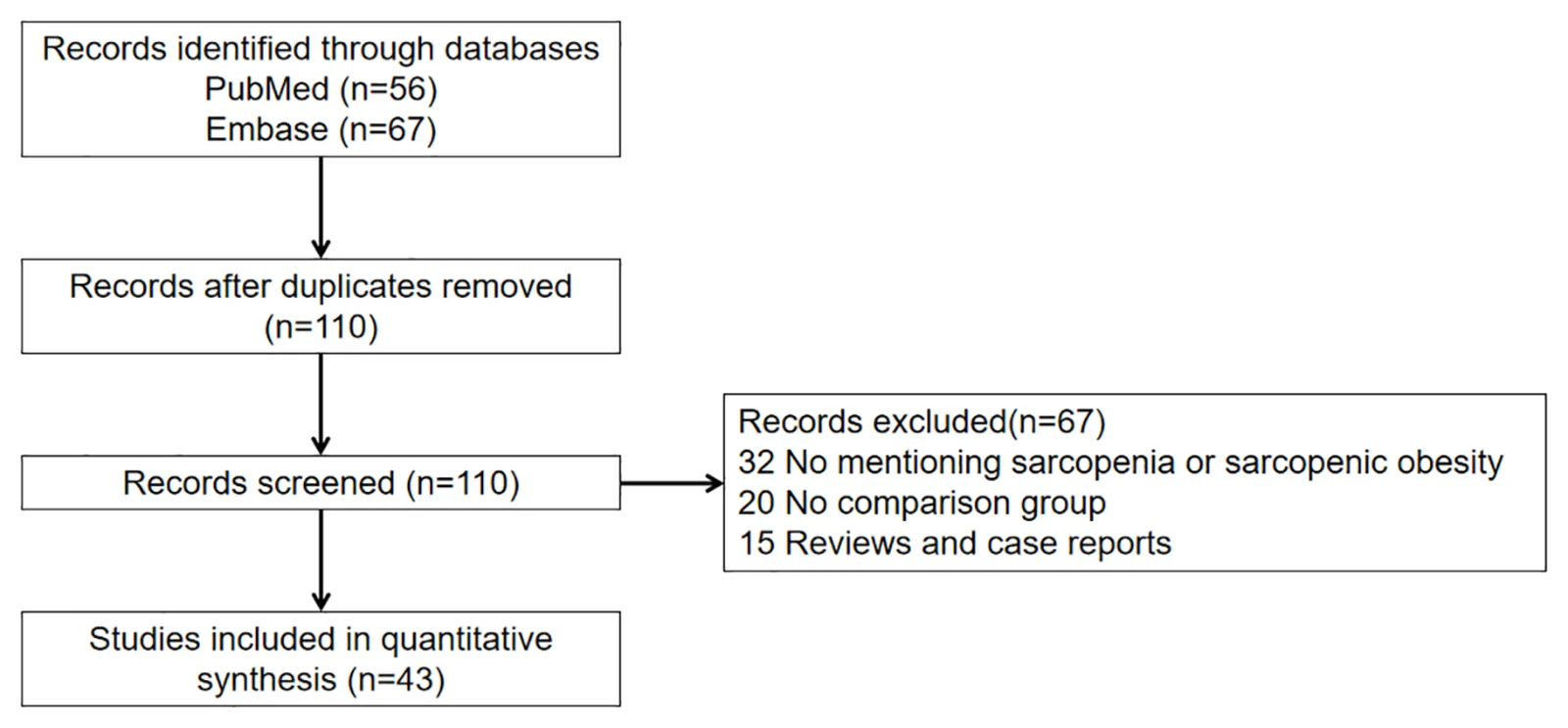
Flow diagram of the study selection.

### 3.2. Basic information of included studies

The basic characteristics of 43 studies are shown in Table 1. The study populations included in this article were from multiple regions worldwide, such as Asia, North America, and Europe, including cross-sectional and retrospective studies. Among them, 38 articles involved sarcopenia and 12 involved SO. The serum biomarkers mentioned included white blood cell count (WBC), interleukins (ILs), hemoglobin (Hb), platelet count, 25-hydroxyvitamin D, albumin (Alb), alkaline phosphatase (ALP), alanine transaminase (ALT), aspartate transaminase (AST), blood urea nitrogen (BUN), C-reactive protein (CRP), serum creatinine, erythrocyte sedimentation rate, blood glucose, thyroid-stimulating hormone (TSH), triglycerides (TGs), low-density lipoprotein cholesterol (LDL-C) and cystatin C etc.

**Table 1:** Basic characteristics of included studies.

First author	Country	Study tpye	Samplesize	Experimental group	Control group	Serum biomarkers
Khalil 2024	Italy	Prospectively	79	Sarcopenia (n = 32)	Non-Sarcopenia (n = 27)	Alb,Cr, Cystatin C,HOMA-IR,25 (OH) Vit D,HDL-C,WBC,HbA1c,CRP,IL-6,LDL-C,Glu,TG,TC
Aldisi 2022	Saudi Arabia	Cross-sectional	76	Sarcopenia (n = 26)	Non-Sarcopenia (n = 50)	HDL,CRP,TC,IL-6,Glu,TNF-ɑ
Can 2016	Turkey	Cross-sectional	72	Sarcopenia (n = 36)	Non-Sarcopenia (n = 36)	Alb, Uric acid, Hb, TG
Chen 2021	China	Cross-sectional	408	Sarcopenia (n = 43)	Non-Sarcopenia (n = 365)	Cr,ALT,TC
Chi-Jen Lo 2023	China	Cross-sectional	120	Sarcopenia (n = 63)	Non-Sarcopenia (n = 57)	Cr,TSH,Alb,WBC,Uric acid,HbA1c,T4,Hb,BUN,HDL,TG,Glu,LDL,TC,Platelet,Vit B12
Dalbeni 2023	Italy	Retrospective study	90	Sarcopenia (n = 55)	Non-Sarcopenia (n = 37)	Cr,WBC,PLT,Hb
Fantin 2024	Spain	Cross-sectiona	34	Sarcopenia (n = 21)	Non-Sarcopenia (n = 13)	HDL-C,LDL-C,Cr, Glu,TC.TG
Hirose 2020^1^	Japan	Cross-sectional	234	Sarcopenia (n = 74)	Non-Sarcopenia (n = 160)	Alb, Hb
Hirose 2020^2^	Japan	Cross-sectional	114	Sarcopenia (n = 37)	Non-Sarcopenia (n = 77)	Alb, Hb
Kameda 2021	Japan	Cross-sectional	19	Sarcopenia (n = 6)	Non-Sarcopenia (n = 13)	Cr, Alb, WBC, HbA1c, ALT, Hb, BUN, AST, HDL, CK, TG, Glu, LDL-C,Platelet
Liu 2020	China	Cross-sectional	3583	Sarcopenia (n = 750)	Non-Sarcopenia (n = 2833)	HDL,TG,LDL-C,TC,Glu,BUN,Insulin,WBC,Vitamin D,Alb,Cr,Uric Acid
Liu 2021	China	Cross-sectional	42	Sarcopenia (n = 18)	Non-Sarcopenia (n = 24)	IL-1ß,IL-6
Lu 2013	China	Cross-sectional	420	Sarcopenia (n = 136)	Non-Sarcopenia (n = 284)	HDL-C,TG,Glu,TC
Montes 2017	Spain	Cross-sectional	116	Sarcopenia (n = 41)	Non-Sarcopenia (n = 75)	Cr,CRP,Alb,ESR,Hb,HDL-C,TGs,Glu,LDL-C,TC,Platelets
Morawin 2023	United States	Cross-sectional	173	Sarcopenia (n = 39)	Non-Sarcopenia (n = 134)	Bilirubin,RBC,WBC,Hb,HDL-C,Glu,LDL-C,TG,TC,Platelets,CRP,Alb,TNF-ɑ,IL-6
Ozturk 2018	Turkey	Cross-sectional	419	Sarcopenia (n = 105)	Non-Sarcopenia (n = 314)	ALP,Alb,CRP,Uric acid,Hb,ALT,AST,ESR
Park 2024	Korea	Cross-sectional	66	Sarcopenia (n = 17)	Non-Sarcopenia (n = 49)	25(OH)D
Ribeiro 2021	Brazil	Prospectively	247	Sarcopenia (n = 86)	Non-Sarcopenia (n = 161)	Insulin,HDL-C,Glu,LDL-C,TG,TC,TNF-ß,IL-10,IL-1ß,IL-6,TNF-ɑ,IL-1ɑ
Santos 2014	Brazil	Cross-sectional	66	Sarcopenia (n = 17)	Non-Sarcopenia (n = 49)	CRP,Insulinaemia,VLDL-C,HDL-C,Glucose,LDL-C,TG, TC
Scott 2018	Australia	Cross-sectional	706	Sarcopenia (n = 110)	Non-Sarcopenia (n = 596)	TG,HDL-C,Glu,25(OH)D
Shibamoto 2023	Japan	Cross-sectional	451	Sarcopenia (n = 110)	Non-Sarcopenia (n = 596)	CRP,Total bilirubin,Alb,Myostatin,Platelet,BUN
Shin 2022	Korea	Cross-sectional	20	Sarcopenia (n = 10)	Non-Sarcopenia (n = 10)	Glucose, ALT,Hb,AST,Alb,HDL-C,LDL-C,TG,TC
Shin 2023	Korea	Cross-sectional	1021	Sarcopenia (n = 176)	Non-Sarcopenia (n = 845)	Cystatin C,CRP,TSH,Myostatin,Alb, HbA1c,Hb,BUN,25(OH)D
Singhal 2019	India	Cross-sectional	100	Sarcopenia (n = 53)	Non-Sarcopenia (n = 47)	Cystatin C
Tang 2020	China	Cross-sectional	384	Sarcopenia (n = 61)	Non-Sarcopenia (n = 323)	Cystatin C,TG,HDL-C,Lymphocyte,LDL-C,CRP,Neutrophil,TC,bilirubin, Glucose,ALT,GGT,AST,Alb,Cr, Alp,Hb,Platelet,Uric acid
Wang 2021^1^	China	Cross-sectional	124	Sarcopenia (n = 51)	Non-Sarcopenia (n = 73)	TG,DBIL,TC,IBIL,Glucose,HbA1c,TBIL,AST,ALT,Cr
Wang 2021^2^	China	Cross-sectional	127	Sarcopenia (n = 38)	Non-Sarcopenia (n = 89)	TG,DBIL,TC,IBIL,Glucose,HbA1c,TBIL,AST,ALT,Cr
Wumaer 2022	China	Cross-sectional	152	Sarcopenia (n = 76)	Non-Sarcopenia (n = 76)	TG,HDL-C,IL-6,IL-4,IL-10,TC,BUN,Glu,TNF-ɑ,Alb,Cr,Hb
Xu 2022	China	Cross-sectional	80	Sarcopenia (n = 40)	Non-Sarcopenia (n = 40)	TG,LDL-C,TC,HbA1c
Yajima 2023	Japan	Cross-sectional	85	Sarcopenia (n = 33)	Non-Sarcopenia (n = 52)	CRP,Alb,Cystatin C,Hb,BUN,TC,Cr,TIBC
Yang 2015	China	Cross-sectional	505	Sarcopenia (n = 100)	Non-Sarcopenia (n = 405)	CRP,TNF-ɑ,IL-6,HDL-C,TG,LDL-C TC
Eguchi 2019	Japan	Cross-sectional	70	Sarcopenia (n = 47)	Non-Sarcopenia (n = 23)	Cystatin C,25(OH)D
Yee 2020	Australia	Cross-sectional	39	Sarcopenia (n = 11)	Non-Sarcopenia (n = 28)	TSH,Glu,Alb,25(OH)D
Yen 2022	China	Cross-sectional	99	Sarcopenia (n = 46)	Non-Sarcopenia (n = 53)	TG,HDL-C,LDL-C,TC,Glu,Alb,Cr
Yin 2022	China	Cross-sectional	2837	Sarcopenia (n = 497)	Non-Sarcopenia (n = 2340)	TG,HDL-C,LDL-C,TSH,TC,Glu,Insulin,25(OH)D,ALT,AST,Alb,IL-10,TNF-ɑ,IL-6
Yoo 2021	Korea	Cross-sectional	83	Sarcopenia (n = 35)	Non-Sarcopenia (n = 48)	AST,ALT,25(OH)D,Alb,ALP
He 2022	China	Cross-sectional	186	Sarcopenia (n = 93)	Non-Sarcopenia (n = 93)	TC,TG,LDL-C,HDL-C
Yu 2021^1^	China	Cross-sectional	77	Sarcopenia (n = 18)	Non-Sarcopenia (n = 61)	Cr,Cystatin C,TNF-ɑ,Alb,IL-6 (pg/ml) Hb,Uric acid
Yu 2021^2^	China	Cross-sectional	133	Sarcopenia (n = 26)	Non-Sarcopenia (n = 107)	Cr,Cystatin C,TNF-ɑ,Alb,IL-6 (pg/ml) Hb,Uric acid
Zou 2024	China	Cross-sectional	1674	Sarcopenia (n = 398)	Non-Sarcopenia (n = 1276)	WBC,Alb,Hb,Platelet
Chang 2023	China	Cross-sectional	816	Sarcopenia (n = 499)	Non-Sarcopenia (n = 317)	Glu,ALT,TBil,AST,Cr,BUN,TC, TG,LDL-C,HDL-C,
Yong He 2022	China	Cross-sectional	4099	Sarcopenia (n = 780)	Non-Sarcopenia (n = 3319)	TSH,Glu,Alb,ALT,AST,Cr,TG,TC,HDL-C,LDL-C,25(OH)D,WBC,PLT
Yang 2015	China	Cross-sectional	466	SO (n = 61)	Healthy individuals (n = 405)	TC,TG,HDL-C,LDL-C,CRP,IL-6,TNF
Ribeiro 2021	Brazil	Prospectively	189	SO (n = 28)	Healthy individuals (n = 1610)	TC,LDL-C,HDL-C,TG,Insulin,Glu,IL,TNF
Fantin 2024	Spain	Cross-sectional	25	SO (n = 12)	Healthy individuals (n = 13)	Glu,TC,LDL-C,HDL-C,TG,Cr
Scott 2018	Australia	Cross-sectional	649	SO (n = 80)	Healthy individuals (n = 569)	25(OH)D,Glu,HDL-C,TG
Lu 2013	China	Cross-sectional	399	SO (n = 115)	Healthy individuals (n = 284)	TC,TG,HDL-C
Liu 2020	China	Cross-sectional	2952	SO (n = 119)	Healthy individuals (n = 2833)	TC,TG,HDL-C (mmol/L)
Khalil 2024	Italy	Prospectively	24	SO (n = 7)	Simple obesity (n = 17)	CRP,IL-6,Glu,Cr,Cystatin C
Nascimento2018	Brazil	Prospectively	64	SO (n = 31)	Simple obesity (n = 33)	TG,HDL-C,LDL-C,Uric acid, Glu
Yang 2014	Taiwan	Cross-sectional	339	SO (n = 61)	Simple obesity (n = 278)	TC,TG,HDL-C,LDL-C,CRP,IL-6,TNF
Ribeiro 2021	Brazil	Prospectively	101	SO (n = 28)	Simple obesity (n = 73)	TC,LDL-C,HDL-C,TG,Glu,Insulin, IL
Kim 2019^1^	South Korea	Cross-sectional	516	SO (n = 287)	Simple obesity (n = 229)	Glu,Insulin,TC,LDL-C,HDL-C,TG,AST,ALT,25(OH)D
Kim 2019^2^	South Korea	Cross-sectional	383	SO (n = 179)	Simple obesity (n = 204)	Glu,Insulin,TC,LDL-C,HDL-C,TG,AST,ALT,25(OH)D
Dutra 2015	Brazil	Cross-sectional	130	SO (n = 27)	Simple obesity (n = 103)	IL-6,CRP,TNF
Yang 2015	China	Cross-sectional	339	SO (n = 61)	Simple obesity (n = 278)	TC,TG,HDL-C,LDL-C,CRP,IL-6,TNF
Fantin 2024	Spain	Cross-sectional	43	SO (n = 12)	Simple obesity (n = 31)	Glu,TC,LDL-C,HDL-C,TG,Cr
Scott 2018	Australia	Cross-sectional	525	SO (n = 80)	Simple obesity (n = 445)	25(OH)D,Glu,TG,HDL-C
Santos 2014	Brazil	Cross-sectional	149	SO (n = 32)	Simple obesity (n = 117)	Glu,CRP,TC,TG,HDL-C,LDL-C,VLDL-C,Insulin
Lu 2013	China	Cross-sectional	180	SO (n = 115)	Simple obesity (n = 65)	TC,TG,Glu
Liu 2020	China	Cross-sectional	917	SO (n = 119)	Simple obesity (n = 798)	Insulin,Glu,TC,TG,HDL-C,LDL-C,Alb,WBC,25(OH)D

### 3.3. Biomarker analysis

#### 3.3.1. Inflammation biomarkers in sarcopenia and SO

As shown in Figure 2 compared with non-sarcopenic patients, sarcopenic patients had elevated serum levels of CRP (SMD: 0.20, 95%CI: 0.10–0.30), ILs (SMD: 0.25, 95%CI: 0.07–0.42), and tumor necrosis factor (TNF) (SMD: 0.18, 95%CI: 0.07–0.30), with no significant differences in WBC (SMD:−0.04, 95%CI:−0.16–0.08) and ESR (SMD: 0.27, 95%CI: −1.43–1.96)(Fig. 3). Further subgroup analyses revealed that in European and American populations, compared with non-sarcopenia patients, sarcopenia patients exhibited no significant differences in serum CRP (SMD: 0.05, 95% CI: 0.10–0.20), ILs (SMD: 0.14, 95% CI:–0.13–0.42), TNF (SMD: 0.01, 95% CI:–0.15–0.16), WBC counts (SMD:–0.37, 95% CI:–0.89–0.15) and ESR (SMD: 0.27, 95% CI:–1.43–1.96). In contrast, among Asian populations, sarcopenia patients demonstrated higher serum CRP levels (SMD: 0.32, 95% CI: 0.19–0.45), ILs (SMD: 0.33, 95% CI: 0.09–0.57), and TNF (SMD: 0.38, 95% CI: 0.22–0.55) compared with non-sarcopenia patients, while no significant differences were observed in WBC counts (SMD: 0.02, 95% CI: −0.05–0.09). Sensitivity analyses were conducted to assess the influence of each individual study on pooled effect sizes. For most inflammatory markers, the direction of pooled estimates remained positive after sequential omission of each study, and the 95%CIs largely overlapped across analyses. Several individual studies could change the magnitude of the pooled effect, yet no single study reversed the overall direction of effect. For ESR, only 3 studies were included, leading to relatively large fluctuations in effect magnitude, although the effect direction remained unchanged. The detailed subgroup results are presented in Supplementary Fig. S1, Supplemental Digital Content 2.

**Figure 2.**
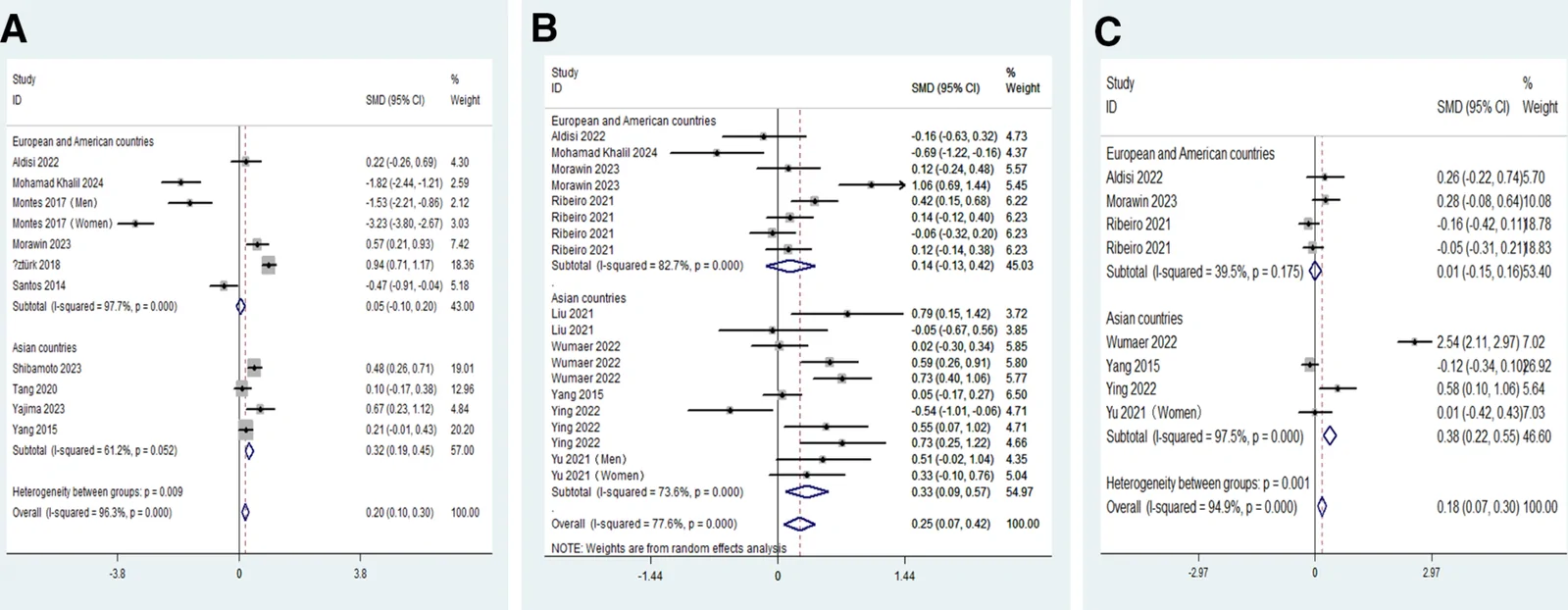
Forest plots of the standardized mean difference for sarcopenic patients and healthy individuals. (A) CRP; (B) ILs; (C) TNF. CI = confidence interval, SMD = standardized mean difference.

**Figure 3.**
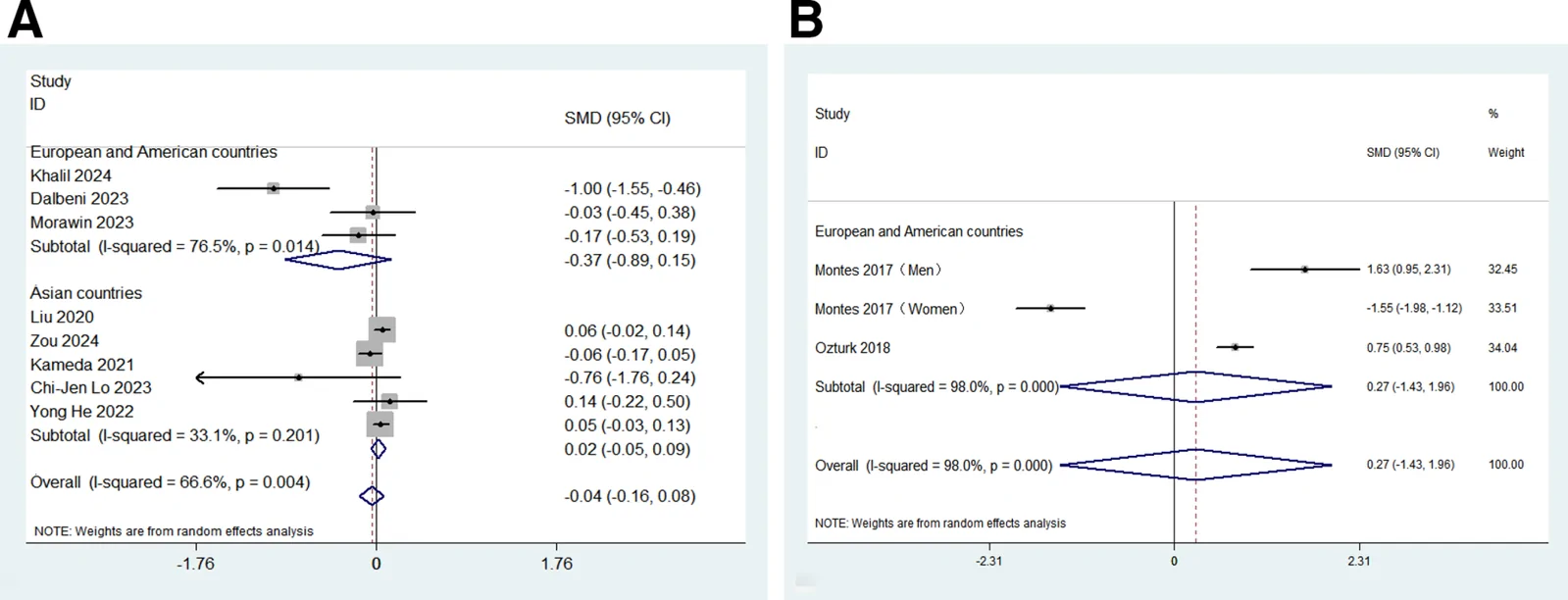
Forest plots of the standardized mean difference for sarcopenic patients and healthy individuals. (A) WBC count; (B) ESR. CI = confidence interval, SMD = standardized mean difference.

As shown in Figure 4, SO patients had higher serum levels of CRP (SMD: 0.67, 95%CI: 0.50–0.84) and WBC count (SMD: 0.71, 95%CI: 0.06–1.36) than healthy individuals. Due to the limited number of studies included, no regional subgroup analysis was performed. Sensitivity analyses were performed to evaluate the influence of each individual study on the pooled effect sizes for CRP and WBC count. After sequentially omitting each study, the direction of pooled effect estimates remained positive without reversal, and the 95%CIs largely overlapped across analyses. Although removal of several studies changed the magnitude of the pooled effect, no single study completely altered the overall conclusion. The detailed subgroup results are presented in Supplementary Fig. S2, Supplemental Digital Content 3.

**Figure 4.**
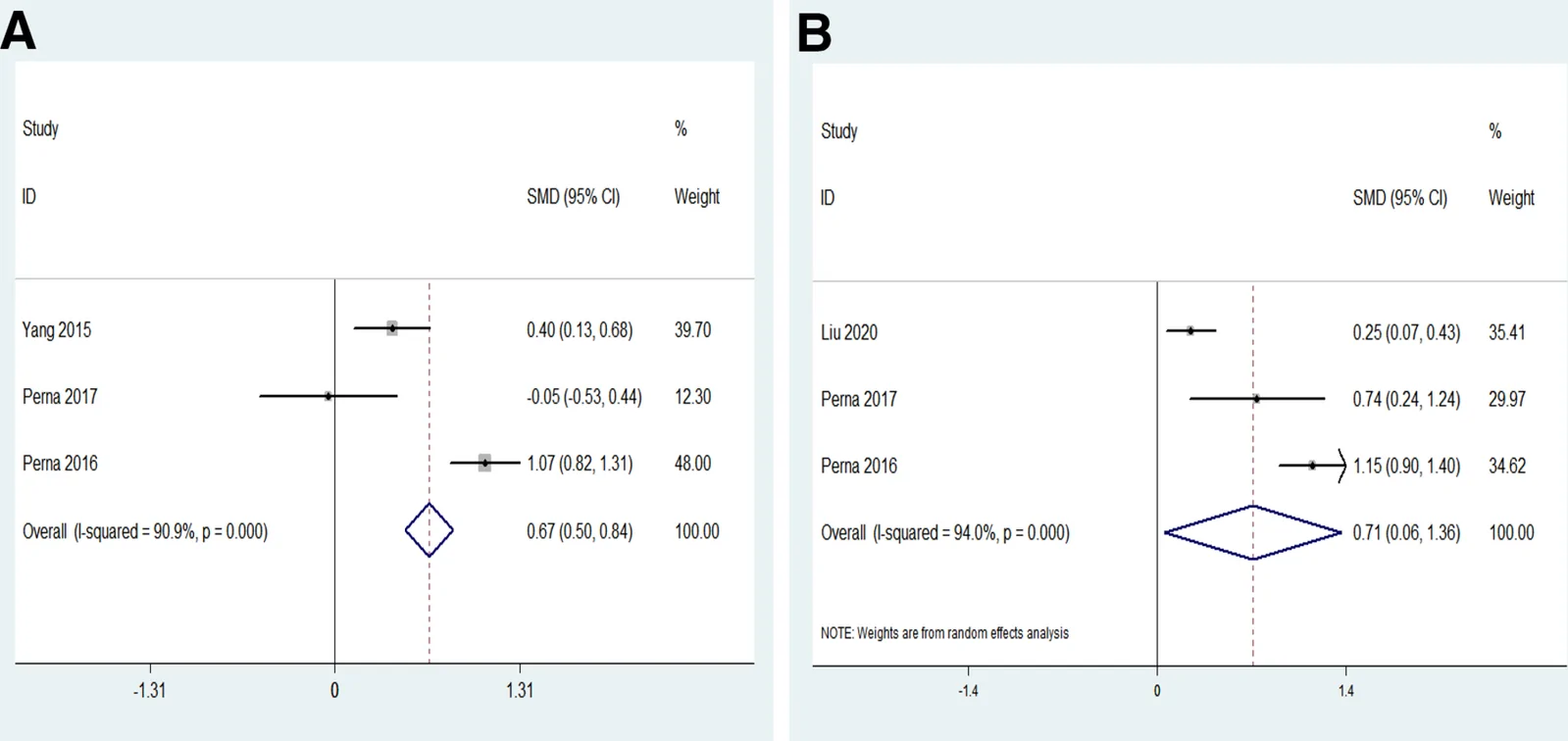
Forest plots of the standardized mean difference for sarcopenic obesity patients and healthy individuals (A) CRP; (B) WBC count. CI = confidence interval, SMD = standardized mean difference.

No significant differences were observed in CRP (SMD: 0.18, 95%CI: −0.02–0.37) and IL-6 levels (SMD: 0.09, 95%CI: −0.08–0.27) between SO patients and those with simple obesity. Sensitivity analyses were performed to assess the influence of each individual study on the pooled effect sizes for CRP and IL-6. After omitting each study sequentially, the direction of pooled effect estimates remained unchanged, and the 95%CIs overlapped substantially across all analyses. No single study was found to dominate the overall pooled results, indicating that our meta-analysis findings for CRP and IL-6 were robust. The detailed subgroup results are presented in Supplementary Fig. S3, Supplemental Digital Content 4.

#### 3.3.2. Metabolic biomarkers in sarcopenia and SO

As shown in Figure 5, sarcopenic patients had elevated serum levels of cystatin C (SMD: 0.77, 95% CI: 0.30–1.25), BUN (SMD: 0.18, 95%CI: 0.09–0.27), and high-density lipoprotein cholesterol (HDL-C) (SMD: 0.19, 95%CI: 0.03–0.34)compared with non-sarcopenic patients, while ALT (SMD: −0.21, 95%CI: −0.35– −0.08), TG (SMD: −0.27, 95%CI: −0.42–−0.12), uric acid (SMD: −0.14, 95%CI: −0.26– −0.03), and blood glucose levels (SMD: −0.21, 95%CI: −0.37–−0.05) were negatively correlated with sarcopenia (Fig. 6). No significant differences were found in AST (SMD: 0.03, 95%CI: −0.03–0.08), ALP (SMD: −0.06, 95%CI: −0.22–0.10), bilirubin (SMD: −0.06, 95%CI: −0.15–0.02), total cholesterol (TC) (SMD: 0.06, 95%CI: −0.08–0.19), creatinine (SMD: 0.10, 95%CI: −0.12–0.32), and LDL-C (SMD: 0.14, 95%CI: −0.03–0.30) levels between sarcopenic and non-sarcopenic patients. The subgroup analysis revealed that in the Asian population, patients with sarcopenia exhibited higher levels of cystatin C (SMD: 0.47,95% CI: 0.13–0.81), BUN (SMD: 0.18,95% CI: 0.09–0.27), and HDL-C (SMD: 0.23,95% CI: 0.12–0.35) compared with non-sarcopenic individuals. The ALT level showed a negative correlation with sarcopenia (SMD: −0.26,95% CI: −0.38– −0.14). In contrast, compared with non-sarcopenic subjects, sarcopenic patients did not demonstrate significant differences in LDL-C (SMD: 0.16,95% CI:–0.02–0.34), TG (SMD:–0.11,95% CI:–0.25–0.02), uric acid (SMD:–0.10,95% CI:–0.20–0.00), blood glucose (SMD: 0.01,95% CI:–0.11–0.14), AST (SMD: 0.02,95% CI:–0.03–0.08), bilirubin (SMD: –0.08,95% CI:–0.17–0.00), TC (SMD: 0.04,95% CI:–0.09–0.17), or creatinine (SMD:–0.03, 95% CI:–0.12–0.07). Among European and American populations, compared with non-sarcopenia individuals, sarcopenia patients exhibited lower levels of TG (SMD: −0.74; 95% CI: −1.27–−0.21) and blood glucose (SMD: −0.68; 95% CI: −1.22–−0.14), whereas no significant differences were observed in serum HDL-C (SMD: 0.17; 95% CI: −0.42–0.75), LDL-C (SMD: −0.03; 95% CI: −0.55–0.50), creatinine (SMD: 0.47; 95% CI: −1.42–2.37), or TC (SMD: −0.01; 95% CI: −0.89–0.87). Since there is limited literature available on the inclusion of cystatin C, ALT, AST, BUN, bilirubin, and uric acid in these populations, no specific subgroup analysis was conducted. Table 2 shows SMD of metabolic biomarkers about the sarcopenia groups and the healthy individuals. Sensitivity analyses were performed to evaluate whether individual studies substantially influenced the overall pooled results for metabolic indicators. After sequentially omitting each single study, the direction and magnitude of the pooled effect sizes for all outcomes (including lipid profiles, glucose, uric acid, liver and renal function indexes) remained stable. The 95%CIs across all leave-one-out analyses overlapped considerably, and no single study caused a reversal of the effect direction or an obvious alteration in statistical significance. Although several indicators had a relatively limited number of included studies, no individual observation dominated the overall pooled estimates. These findings demonstrated that our meta-analytic results were statistically robust and reliable (Supplementary Fig. S4–S7, Supplemental Digital Content 5).

**Table 2:** SMD of metabolic biomarkers about the sarcopenia groups and the healthy individuals.

	Asian	European or American
CystatinC	0.47 (0.13–0.81)	–
BUN	0.18 (0.09–0.27)	–
HDL-C	0.23 (0.12–0.35)	0.17 (-0.42–0.75)
ALT	−0.26 (−0.38–−0.14)	–
LDL-C	0.16 (−0.02–0.34)	−0.03 (−0.15–0.50)
TG	−0.11 (−0.25–0.02)	−0.74 (−1.27–−0.21)
Uric acid	−0.10 (−0.20–0.00)	–
Blood glucose	0.01 (−0.11–0.14)	−0.68 (−1.22–−0.14)
AST	0.02 (−0.03–0.08)	–
Bilirubin	−0.08 (−0.17–0.00)	–
TC	0.04 (−0.09–0.17)	−0.01 (−0.89–0.87)
Creatinine	−0.03 (−0.12–0.07)	0.47 (−1.42–2.37)

ALT = alanine transaminase, AST = aspartate transaminase, BUN = blood urea nitrogen, HDL-C = high-density lipoprotein cholesterol, LDL-C = low-density lipoprotein cholesterol, TC = total cholesterol, TG = triglyceride.

**Figure 5.**
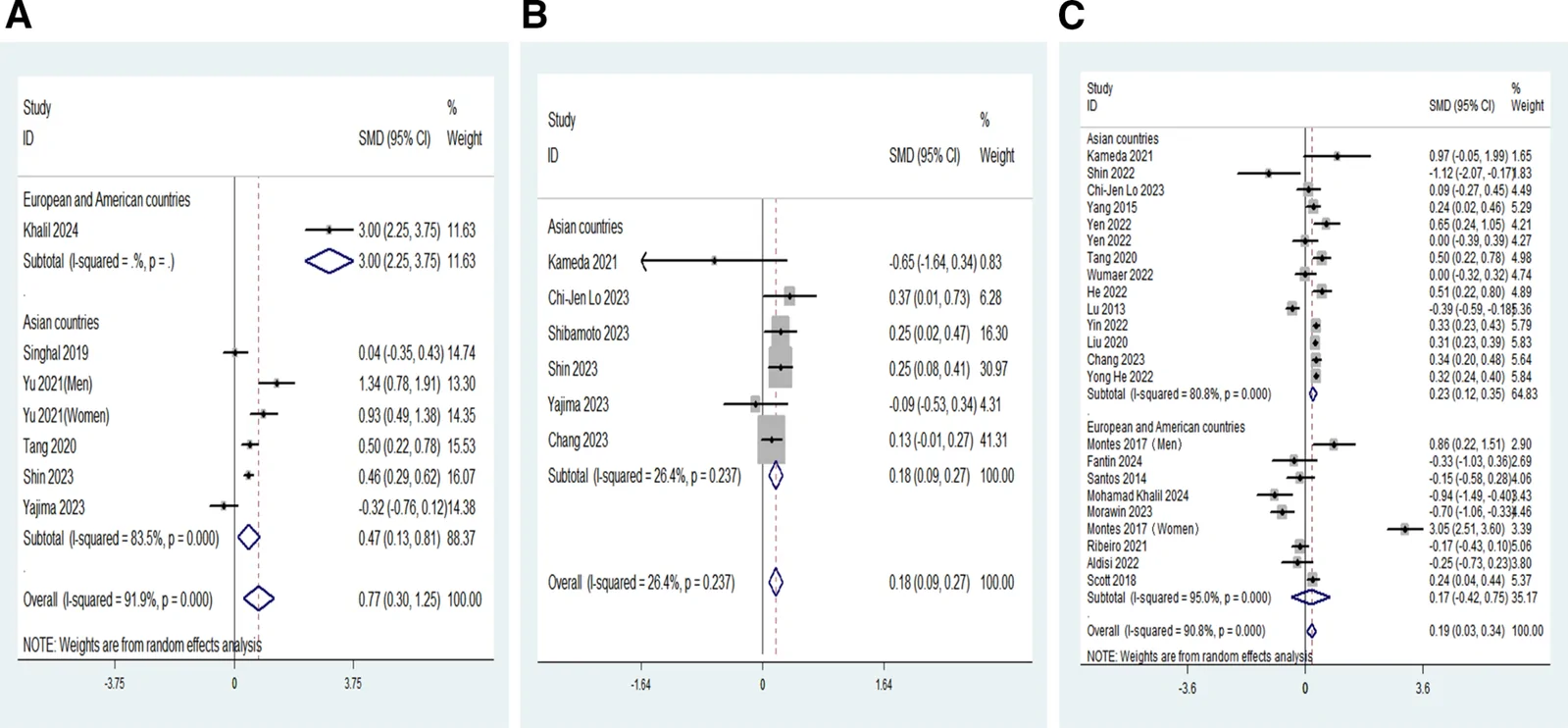
Forest plots of the standardized mean difference for sarcopenic patients and non-sarcopenic patients (A) Cystatin C; (B) BUN; (C) HDL-C. CI = confidence interval, SMD = standardized mean difference.

**Figure 6.**
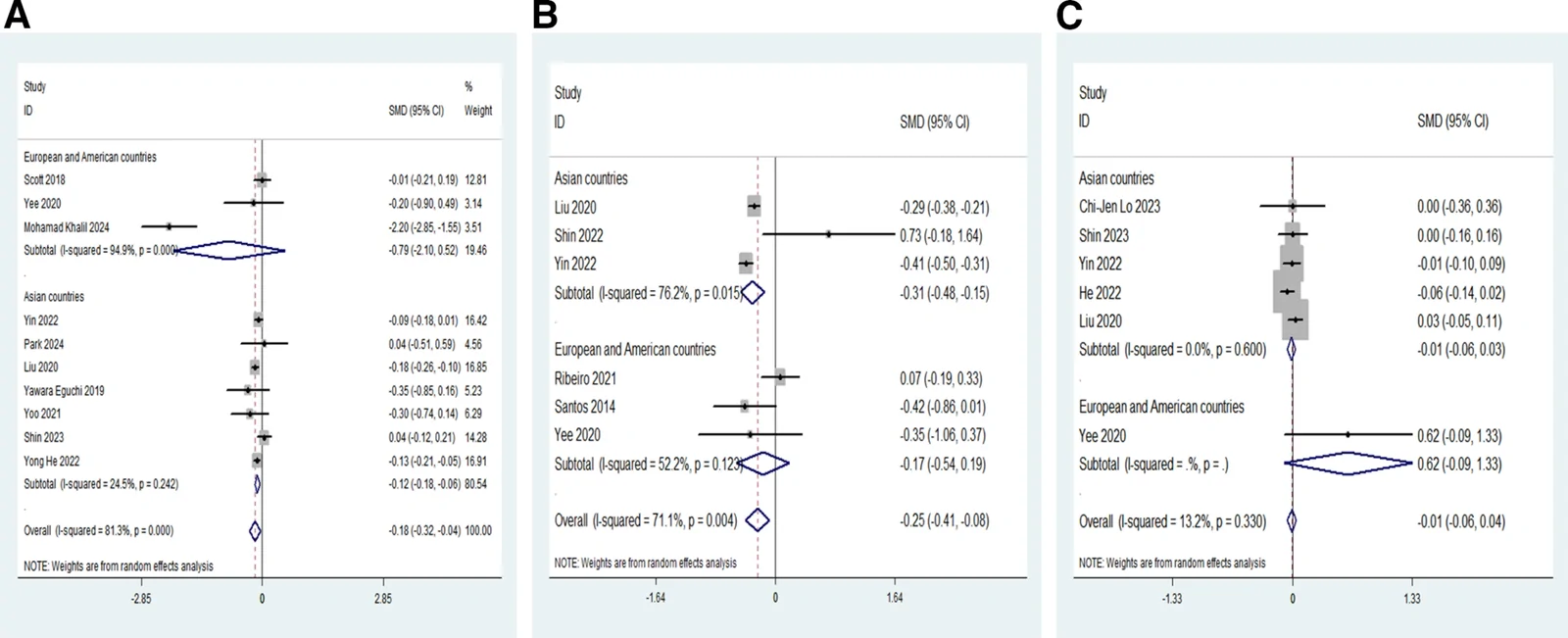
Forest plots of the standardized mean difference for sarcopenia compared with non-sarcopenia (A) Vitamin D; (B) insulin; (C) TSH. CI = confidence interval, SMD = standardized mean difference, TSH = thyroid-stimulating hormone.

SO patients had higher serum levels of LDL-C (SMD: 0.25, 95%CI: 0.11–0.39), TC (SMD: 0.21, 95%CI: 0.09–0.33), TG (SMD: 0.22, 95%CI: 0.11–0.32), creatinine (SMD: 0.30, 95%CI: 0.03–0.56), uric acid (SMD: 0.47, 95%CI: 0.33–0.61), and than healthy individuals, while HDL-C (SMD: −0.29, 95%CI: −0.39–−0.18) was negatively correlated with SO. Blood glucose levels (SMD: 0.27, 95%CI: −0.01–0.55) showed no difference between the 2 groups. Subgroup analysis revealed that among Asian populations, patients with SO exhibited higher serum levels of LDL-C (SMD: 0.29; 95% CI: 0.14–0.44), TC (SMD: 0.22; 95% CI: 0.10–0.35), and TG (SMD: 0.18; 95% CI: 0.06–0.31) compared to healthy individuals, whereas HDL-C (SMD: −0.39; 95% CI: −0.52–−0.27) were lower than those in healthy individuals; no significant difference was observed in blood glucose levels between the 2 groups (SMD: 0.42; 95% CI: −0.07–0.90). In contrast, among European and American populations, patients with SO demonstrated higher TG (SMD: 0.31; 95% CI: 0.11–0.50) and creatinine (SMD: 0.44; 95% CI: 0.23–0.65) levels compared to healthy individuals; however, no significant differences were observed in LDL-C (SMD: 0.03; 95% CI: −0.33–0.39), TC (SMD: 0.12; 95% CI: −0.24–0.48), blood glucose (SMD: 0.08; 95% CI: −0.12–0.28), or HDL-C (SMD: −0.03; 95% CI: −0.22–0.17) levels between the 2 groups. Further sensitivity analyses demonstrated that the results were statistically robust and reliable (Supplementary Fig. S8–S9, Supplemental Digital Content 6).

SO patients had lower TG levels (SMD: −0.14, 95%CI: −0.26–−0.02) than those with simple obesity, with no significant differences in HDL-C (SMD: 0.02, 95%CI: −0.06–0.10), LDL-C (SMD: −0.02, 95%CI: −0.10–0.07), TC (SMD: 0.06, 95%CI: −0.08–0.20), and blood glucose levels (SMD: 0.07,95%CI: −0.08–0.22) between the 2 groups. Further subgroup analyses revealed that among Asian populations, SO patients exhibited higher blood glucose levels than patients with simple obesity (SMD: 0.14, 95%CI: 0.02–0.26), whereas no significant intergroup differences were observed in TG (SMD: −0.09, 95%CI: −0.29–0.10), HDL-C (SMD: −0.02, 95%CI: −0.11–0.08), LDL-C (SMD: −0.01, 95%CI: −0.10–0.09), and TC (SMD: 0.11, 95%CI: −0.05–0.26). In European and American populations, SO patients had lower TG levels relative to patients with simple obesity (SMD: −0.19, 95%CI: −0.38–−0.01). No significant differences were found in HDL-C (SMD: 0.08, 95%CI: −0.13–0.29), LDL-C (SMD: −0.06, 95%CI: −0.30–0.17), TC (SMD: −0.13, 95%CI: −0.40–0.13), and blood glucose levels (SMD: −0.01, 95%CI: −0.32–0.30) between the 2 groups. As shown in Supplementary Fig. S10, Supplemental Digital Content 7, sensitivity analyses were performed for TG, TC, LDL‑C, HDL‑C and blood glucose. Sequentially omitting each individual study did not substantially change the direction and magnitude of the pooled effect estimates for HDL‑C, LDL‑C, TC and blood glucose, with overlapping 95% CIs across all exclusion models, indicating that these pooled findings were robust. For TG, the overall pooled effect remained consistently negative after excluding each study in turn, and no single study reversed the statistical significance of the result, which also suggested the stability of the pooled association.

#### 3.3.3. Hormonal biomarkers in sarcopenia and SO

As shown in Figure 6, higher serum levels of vitamin D (SMD: −0.18, 95%CI: −0.32–−0.04) and insulin (SMD: −0.25, 95%CI: −0.41–−0.08) were associated with a lower incidence of sarcopenia compared with non-sarcopenia, with no significant difference in TSH (SMD: −0.01, 95%CI: −0.06–0.04) levels between the 2 groups.

Subgroup analyses stratified by geographical region showed that higher levels of vitamin D (SMD: −0.12, 95%CI: −0.18–−0.06) and insulin (SMD: −0.31, 95%CI: −0.48–−0.15) were associated with a lower prevalence of sarcopenia in Asian populations. By contrast, no significant differences in vitamin D (SMD: −0.79, 95%CI: −2.10–0.52) and insulin (SMD: −0.17, 95%CI: −0.54–0.19) were observed between individuals with sarcopenia and healthy controls in European and American populations. Sensitivity analyses for vitamin D, TSH and insulin demonstrated stable pooled estimates with consistent effect directions and overlapping 95% CIs after omitting each study sequentially, suggesting reliable results (Supplementary Fig. S11, Supplemental Digital Content 8).

Insulin levels (SMD: 0.17, 95%CI: 0.01–0.34) were positively correlated with SO, while vitamin D levels (SMD: −0.24, 95%CI: −0.39–−0.10) were negatively correlated with SO compared with healthy individuals. No significant difference in vitamin D levels (SMD: −0.03, 95%CI: −0.13–0.07) was observed between SO patients and those with simple obesity. Due to the limited number of included studies, no subgroup analyses or sensitivity analyses were conducted based on geographic region.

#### 3.3.4. Hematological biomarkers in sarcopenia and SO

As shown in Figure 7, Alb (SMD: −0.45, 95%CI: −0.57–−0.34) and Hb (SMD: −0.51, 95%CI: −0.73–−0.29) levels were negatively correlated with sarcopenia compared with non-sarcopenia, with no significant difference in platelet count. No data on hematological biomarkers associated with SO were available.

**Figure 7.**
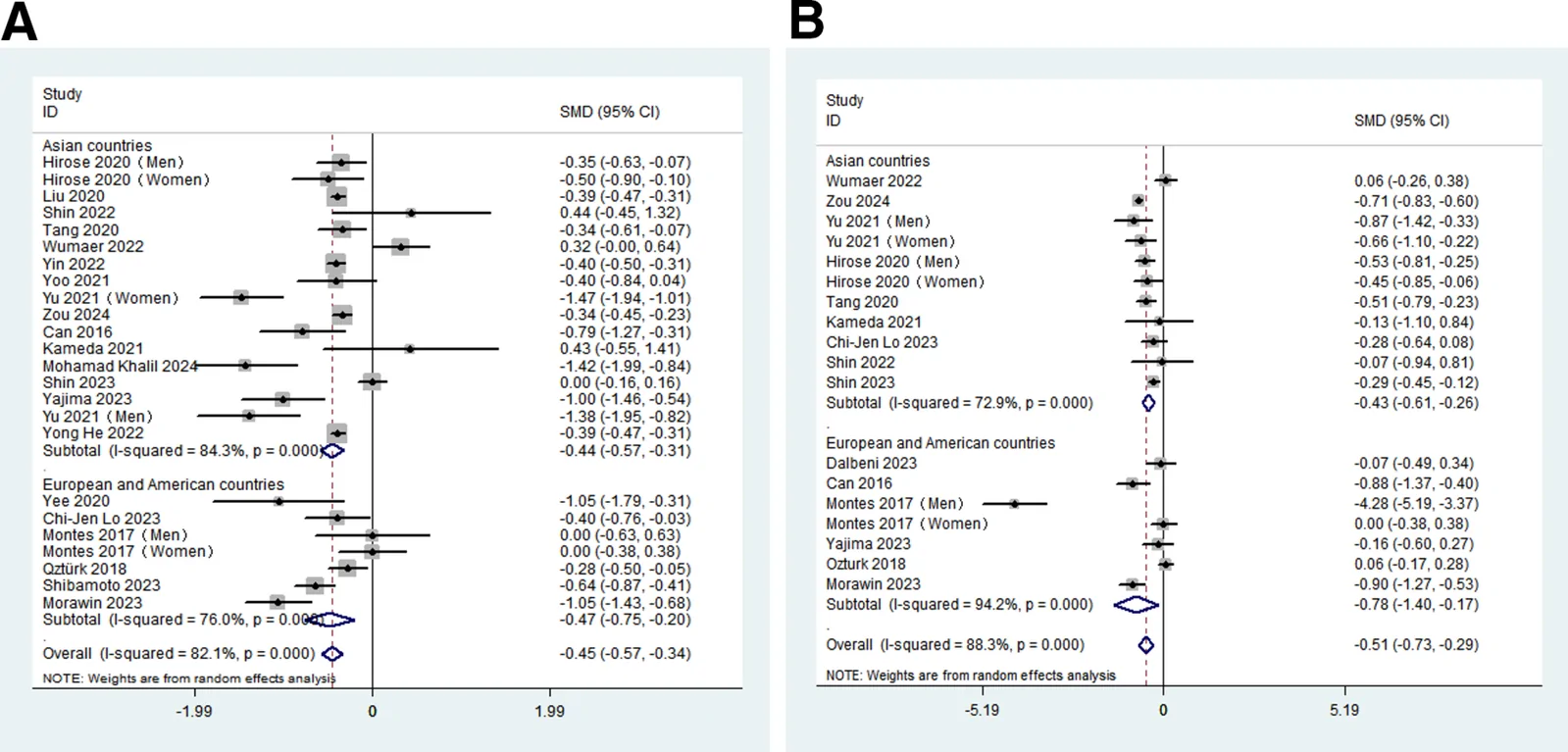
Forest plots of the standardized mean difference for sarcopenia compared with non-sarcopenia (A) Alb; (B) Hb. CI = confidence interval, SMD = standardized mean difference.

Further subgroup analyses demonstrated that higher levels of Alb (SMD: −0.44, 95%CI: −0.57–−0.31) and Hb (SMD: −0.43, 95%CI: −0.61–−0.26) were associated with a lower prevalence of sarcopenia in Asian populations, and this consistent association was also observed in European and American populations (Alb: SMD: −0.47, 95%CI: −0.75–−0.20) and Hb (SMD: −0.78, 95%CI: −1.40–−0.17). Sensitivity analyses demonstrated that the pooled SMDs for Alb and Hb (Supplementary Fig. S12, Supplemental Digital Content 9) remained directionally consistent after sequential exclusion of each study, with no single study dominating the overall pooled estimates, supporting the robustness of our results (Supplementary Fig. S12, Supplemental Digital Content 9).

## 4. Discussion

Previous studies have shown that sarcopenia and SO have complex similarities and significant pathophysiological differences in various biomarkers, including inflammatory, metabolic, hematological, and hormonal markers. However, the underlying mechanisms of sarcopenia and SO remain unclear, and the types of serum biomarkers investigated in relevant studies are limited, requiring further validation by numerous studies. In this study, we extracted the levels of various serum biomarkers from the included articles and used meta-analysis to compare sarcopenia with non-sarcopenia, SO with healthy individuals, and SO with simple obesity, aiming to explore the correlations between different serum biomarker levels and sarcopenia/SO. All potential biomarkers were included and analyzed using statistical software, which provides certain guiding significance for clinicians in the diagnosis of relevant patients.

In recent years, studies have indicated that the development of SO is closely related to multiple mechanisms such as chronic low-grade inflammation, insulin resistance, oxidative stress, and mitochondrial dysfunction.^[55]^ Systemic inflammatory response is considered a key driving factor for the occurrence of SO. Inflammation-related indicators such as CRP is positively correlated with SO. Elevated inflammatory levels can accelerate skeletal muscle protein catabolism, promote fat deposition, and further lead to the decline in muscle mass and function.^[56]^

Mitochondrial dysfunction plays a core role in the pathogenesis of SO. SO patients exhibit impaired skeletal muscle mitochondrial bioenergetics, reduced ATP production, decreased muscle synthesis capacity, myofiber atrophy, and lower exercise tolerance.^[57]^ Both animal experiments and clinical studies have shown that SO-related abnormalities in mitophagy, mitochondrial dynamics imbalance (abnormal fusion/fission), and energy metabolism disorder are key links in muscle mass loss and fat accumulation.^[58]^ In addition, imbalanced protein metabolism, insufficient amino acid supply, and abnormal fatty acid metabolism are also involved in the development of SO. Specific amino acids (e.g., glutamine, branched-chain amino acids, lysine, methionine) are closely related to muscle metabolism and are considered potential biomarkers and intervention targets.^[59]^ In terms of inflammatory mediators, CRP levels were positively correlated with both sarcopenia and SO. ILs and TNF levels were positively correlated with sarcopenia; however, the number of articles investigating the correlations between ILs/TNF and SO was too small to conduct relevant analyses. WBC had no significant correlation with sarcopenia but was positively correlated with SO. Inflammation is an adaptive response to tissue dysfunction or homeostatic imbalance and the basis of various physiological and pathological processes. Previous studies have shown that persistent tissue dysfunction in the elderly leads to a state of chronic inflammation, characterized by mild elevations in serum nonspecific inflammatory markers such as TNF-α, CRP, and IL-6. Chronic low-grade inflammation can affect the synthesis and catabolism of muscle protein through signal transduction, leading to the reduction in muscle mass, strength, and function, and inducing sarcopenia.^[60]^ The development of both sarcopenia and SO is closely related to chronic low-grade inflammation, in which IL-6 and tumor necrosis factor-α (TNF-α) are core proinflammatory factors, and CRP is a key marker reflecting systemic inflammatory burden. Through a synergistic inflammatory network, the 3 jointly mediate muscle atrophy, fat deposition, and metabolic disorder, forming a vicious circle.^[61]^ IL-6 is mainly secreted by adipose tissue, macrophages, and damaged muscle cells, and promotes the expression of muscle atrophy-related ubiquitin ligases (MuRF1, Atrogin-1) by activating the JAK2-STAT3 pathway, accelerating muscle protein degradation; at the same time, it inhibits the proliferation and differentiation of muscle satellite cells, reducing muscle regenerative capacity. TNF-α inhibits the PI3K/Akt/mTOR signal by activating the NF-κB pathway, reducing muscle protein synthesis, and inducing oxidative stress, mitochondrial damage, and muscle cell apoptosis, further exacerbating muscle loss.^[62]^ CRP is largely synthesized by the liver under the stimulation of IL-6 and TNF-α, and its elevated level not only reflects the degree of systemic inflammation but also directly inhibits insulin signaling, aggravates insulin resistance, and further suppresses muscle synthesis. In SO, visceral fat and intramuscular adipose tissue infiltration are significantly enhanced, leading to increased release of IL-6 and TNF-α and higher CRP levels, forming a vicious circle of “fat-inflammation-muscle atrophy-further fat deposition,” which results in more severe muscle mass loss, muscle weakness, and metabolic abnormalities.^[63]^ In summary, IL-6, TNF-α, and CRP jointly constitute the core inflammatory mechanism of sarcopenia and SO, driving disease progression by regulating protein synthesis-catabolism imbalance, muscle satellite cell dysfunction, insulin resistance, and oxidative stress.

WBC, as a basic indicator of systemic inflammation, has significant differences in its association with sarcopenia and SO. Simple sarcopenia is dominated by muscle loss with relatively normal fat content, and inflammation is mostly confined to the muscle microenvironment, characterized by elevated local cytokines (IL-6, TNF-α), with no obvious changes in WBC,^[64]^ thus showing no significant correlation between them. SO presents a stronger state of systemic low-grade inflammation due to visceral fat accumulation, intramuscular adipose tissue infiltration, and activation of the fat-muscle inflammatory axis. Visceral adipose tissue can secrete a large amount of free fatty acids (FFAs), activate mononuclear-macrophages through the TLR4-MyD88 pathway, and promote the release of neutrophils and lymphocytes from the bone marrow, leading to an increase in peripheral blood WBC; at the same time, IL-6 and TNF-α secreted by adipose tissue can further stimulate the liver to synthesize CRP and amplify the systemic inflammatory response, resulting in a sustained increase in WBC levels.^[64]^ In addition, SO is often accompanied by insulin resistance, enhanced oxidative stress, and metabolic disorder, which further promote the activation and proliferation of inflammatory cells, making WBC levels significantly positively correlated with muscle mass, fat mass, and inflammatory degree. Therefore, WBC count can be used as an important indicator of systemic inflammatory burden in SO, but has limited value in the evaluation of simple sarcopenia.

This study found that there were differences in lipid-related serum biomarkers between sarcopenia and SO patients. Sarcopenic patients had elevated HDL-C levels and reduced TG levels, with no significant correlation between TC/LDL-C levels and the occurrence of sarcopenia. In contrast, SO patients had positive correlations between LDL-C, TC, TG levels and SO, and a negative correlation between HDL-C levels and SO. SO patients had lower TG levels than those with simple obesity, with no significant differences in HDL, LDL, and TC levels between the 2 groups. Muscle mass loss may lead to impaired lipid oxidation capacity, and the body compensates for this by inhibiting the secretion of hepatic very-low-density lipoprotein (VLDL) to reduce systemic TG accumulation. On the contrary, in SO, insulin resistance and chronic inflammation caused by visceral fat accumulation may play a dominant role, leading to enhanced lipolysis and excessive hepatic VLDL production. In simple sarcopenia, patients are characterized by muscle mass loss and normal or low fat content, without visceral fat accumulation and mild systemic low-grade inflammation. Although muscle loss leads to decreased insulin sensitivity, limited FFA release due to less adipose tissue results in reduced hepatic TG synthesis and VLDL secretion, thus decreasing serum TG levels; at the same time, insufficient raw materials for cholesterol synthesis leads to no obvious elevation in TC and LDL-C. In addition, simple sarcopenic patients secrete fewer inflammatory factors from adipose tissue, resulting in weaker inhibition of HDL-C, and muscle atrophy can compensatorily promote hepatic HDL-C synthesis to maintain reverse cholesterol transport, thus relatively increasing HDL-C levels. In SO, patients have both muscle loss and visceral fat accumulation, forming a vicious circle of the “fat-muscle inflammatory axis.” Visceral fat releases a large amount of FFA, stimulating the liver to synthesize TG and VLDL, leading to a significant increase in serum TG; at the same time, a large amount of inflammatory factors (IL-6, TNF-α, CRP) are secreted, inhibiting HDL-C synthesis and accelerating its decomposition, resulting in decreased HDL-C levels; in addition, aggravated insulin resistance and disordered cholesterol metabolism lead to elevated TC and LDL-C levels. Therefore, SO presents a typical atherogenic lipid profile, that is, elevated TC, LDL-C, and TG, and decreased HDL-C. In summary, simple sarcopenia is characterized by decreased TG and elevated HDL-C due to low fat content and mild inflammation, while SO presents atherogenic dyslipidemia due to visceral fat accumulation, systemic inflammation, and insulin resistance. The differences in lipid profiles between the 2 reflect the different pathological states of the fat-muscle-inflammation-metabolism axis.^[65,66]^

Skeletal muscle is a target organ of vitamin D, and the mechanisms underlying the association between vitamin D and sarcopenia/SO have not been fully elucidated. However, the following possible mechanisms have been proposed: First, evidence indicates that vitamin D status can regulate skeletal muscle mitochondrial function; vitamin D deficiency significantly reduces mitochondrial complex I activity and inhibits key genes involved in mitochondrial biogenesis. This impaired mitochondrial function leads to decreased energy metabolism efficiency, manifested as reduced overall energy expenditure, which in turn exacerbates muscle atrophy and fat accumulation. Skeletal muscle is the largest component of systemic energy expenditure. Second, vitamin D can regulate muscle mitochondrial function and biogenesis through the highly coordinated regulation of gene expression by the vitamin D receptor (VDR), thereby affecting changes in body composition. In a mouse model with muscle-specific VDR gene knockout, mitochondrial density is reduced by 20% to 30%, and the activity of the key fatty acid oxidase HAD is decreased by 40%, indicating that the VDR signaling pathway plays a core role in maintaining muscle metabolic homeostasis.^[55]^ Third, vitamin D deficiency may reduce immune, antioxidant, and anti-inflammatory capacities, and affect the ability of adipose tissue to store fatty acids, while FFAs can damage the physiological metabolic environment of peripheral muscle tissue.^[67]^ Notably, clinical intervention trials have further confirmed that vitamin D supplementation can significantly increase appendicular skeletal muscle mass and grip strength in elderly subjects, suggesting that improving vitamin D status may break the vicious circle of sarcopenia and skeletal muscle mass loss by restoring mitochondrial oxidative capacity.

Cystatin C and BUN levels were positively correlated with sarcopenia, while ALT, albumin, and uric acid levels were negatively correlated with sarcopenia. AST, ALP, bilirubin, creatinine, and TSH levels were not associated with sarcopenia. Creatinine and uric acid levels were positively correlated with SO. The possible reasons for these findings are as follows: Cystatin C is a sensitive indicator of early renal injury. Sarcopenia is essentially a state of low-grade chronic inflammation, and inflammation damages renal tubular endothelial cells, leading to decreased early renal filtration function and elevated cystatin C. Enhanced muscle catabolism results in increased production of BUN, and impaired excretion leads to elevated BUN. ALT is abundantly present in skeletal muscle; muscle mass loss in sarcopenic patients leads to reduced intracellular enzyme release and a physiological decrease in serum ALT levels. Sarcopenic patients often have poor appetite and insufficient protein intake; at the same time, chronic inflammation accelerates albumin decomposition, leading to hypoalbuminemia. Skeletal muscle is the main site of purine metabolism; a sharp reduction in muscle mass leads to decreased purine substrates and thus natural reduction in uric acid production. In SO patients, although muscle mass is reduced, insulin resistance caused by obesity leads to glomerular hyperfilt ration injury and decreased renal function, resulting in reduced creatinine excretion. The impact of excretion disorder outweighs the reduction in production, thus increasing creatinine levels. At the same time, insulin resistance in SO patients impairs renal uric acid excretion, and adipose tissue promotes purine synthesis, leading to hyperuricemia.

Fasting blood glucose and fasting insulin levels were positively correlated with SO but negatively correlated with sarcopenia. SO patients have insulin resistance caused by visceral fat accumulation and reduced glucose uptake due to muscle mass loss, and the body is forced to secrete a large amount of insulin (hyperinsulinemia). In contrast, patients with simple sarcopenia are mostly frail with poor food intake, insufficient carbohydrate intake, no fat accumulation, and good insulin sensitivity, thus having low blood glucose and insulin levels.

Sarcopenic patients had decreased albumin and Hb levels, with no significant difference in platelet count compared with non-sarcopenic patients. Skeletal muscle stores 60% of the body’s protein; massive muscle loss in sarcopenic patients inevitably leads to a decrease in albumin, which serves as a circulating protein reserve. At the same time, albumin is a negative acute-phase protein synthesized by the liver, and its synthesis depends on sufficient protein intake and energy. Sarcopenic patients are often accompanied by anorexia and decreased digestive and absorption functions, leading to insufficient intake of amino acids (especially branched-chain amino acids). Insufficient raw materials prevent the liver from synthesizing enough albumin, and muscle protein is also decomposed for energy, forming a vicious circle.^[68]^ In addition, sarcopenia is accompanied by a chronic inflammatory state, and inflammatory factors can inhibit the transcription of albumin mRNA in the liver and promote muscle protein decomposition through the ubiquitin-proteasome pathway. Sarcopenic elderly individuals often have insufficient iron intake and absorption disorders; Hb synthesis requires iron, and iron deficiency leads to decreased Hb and iron deficiency anemia. Sarcopenic patients usually have chronic inflammation, which induces an increase in hepcidin in this state. Hepcidin blocks intestinal iron absorption and sequesters iron in macrophages, leading to functional iron deficiency and decreased Hb.^[69]^ When Hb is low, the oxygen-carrying capacity of the blood is poor, resulting in muscle tissue hypoxia. Hypoxia inhibits myoblast differentiation, promotes myocyte apoptosis, and reduces mitochondrial function, directly exacerbating sarcopenia.

In summary, pure sarcopenia and SO are not different severity forms of the same disease, but rather 2 distinct conditions with independent serum biomarker profiles and pathological mechanisms. The differential expression of their serum biomarkers precisely reflects 2 distinct pathological states: pure muscle loss without fat accumulation, and fat accumulation accompanied by muscle atrophy. This study comprehensively elucidates the correlations and underlying mechanisms between various serum biomarkers and these 2 conditions, advancing the molecular pathophysiological understanding of sarcopenia and SO, and providing reliable evidence-based insights for clinical differentiation, disease severity assessment, and development of personalized intervention strategies.

## 5. Limitation

This study has several limitations. Firstly, there are relatively limited included literature on SO, which leads to insufficient comprehensive data analysis. Secondly, the study population has differences in demographic aspects, such as an unbalanced male-to-female ratio, racial diversity among the included population, differences in the average age of the included population, and variations in the presence or absence of underlying diseases. The majority of the sarcopenia biomarkers discovered in this study were derived from venous blood samples, rather than the more common muscle biopsy. The detection results of biomarkers collected at different time points may vary due to fluctuations in levels, thereby affecting the accurate assessment of their true diagnostic and prognostic values. The measurement tools used in different studies for assessing muscle mass may also contribute to this bias. More clinical studies on sarcopenia and SO are needed in the future to explore this issue.

## Acknowledgments

We gratefully thank all investigators of the included primary studies for providing valuable data. We are grateful to the colleagues for valuable suggestions on study design, literature screening, data extraction and statistical analysis.

## Author contributions

**Data curation:** Yaxian Yang, Chunyu Wang, Xiaoai Chen.

**Methodology:** Yaxian Yang, Wang Xuan, Ruiyuan Xu.

**Software:** Yaxian Yang, Chunyu Wang, Xiaoai Chen.

**Conceptualization:** Wang Xuan.

**Resources:** Ruiyuan Xu.

**Writing – original draft:** Yaxian Yang.

**Writing – review & editing:** Yaxian Yang, Wang Xuan, Ruiyuan Xu, Xiaoai Chen.



























## References

[1] Khan SS Singer BD Vaughan DE. Molecular and physiological manifestations and measurement of aging in humans. Aging Cell. 2017;16:624–33. 10.1111/acel.12601 PMC5506433 28544158

[2] Candow DG Chilibeck PD Forbes SC Fairman CM Gualano B Roschel H. Creatine supplementation for older adults: focus on sarcopenia, osteoporosis, frailty and cachexia. Bone. 2022;162:116467. 10.1016/j.bone.2022.116467 35688360

[3] Choi KM. Sarcopenia and sarcopenic obesity. Korean J Intern Med. 2016;31:1054–60. 10.3904/kjim.2016.193 PMC5094937 27809450

[4] Gao Q Mei F Shang Y. Global prevalence of sarcopenic obesity in older adults: a systematic review and meta-analysis. Clin Nutr. 2021;40:4633–41. 10.1016/j.clnu.2021.06.009 34229269

[5] Mirzai S Carbone S Batsis JA Kritchevsky SB Kitzman DW Shapiro MD. Sarcopenic obesity and cardiovascular disease: an overlooked but high-risk syndrome. Curr Obes Rep. 2024;13:532–44. 10.1007/s13679-024-00571-2 PMC11306598 38753289

[6] Karanth SD Washington C Cheng T-YD. Inflammation in relation to sarcopenia and sarcopenic obesity among older adults living with chronic comorbidities: results from the National Health and Nutrition Examination Survey 1999–2006. Nutrients. 2021;13:3957. 10.3390/nu13113957 PMC8621174 34836213

[7] Liberati A Altman DG Tetzlaff J. The PRISMA statement for reporting systematic reviews and meta-analyses of studies that evaluate health care interventions: explanation and elaboration. Ann Intern Med. 2009;151:W–65. 10.7326/0003-4819-151-4-200908180-00136 19622512

[8] Chen L-K Woo J Assantachai P. Asian Working Group for sarcopenia: 2019 consensus update on sarcopenia diagnosis and treatment. J Am Med Dir Assoc. 2020;21:300–7.e2. 10.1016/j.jamda.2019.12.012 32033882

[9] Cruz-Jentoft AJ Bahat G Bauer J; Writing Group for the European Working Group on Sarcopenia in Older People 2 (EWGSOP2), and the Extended Group for EWGSOP2. Sarcopenia: revised European consensus on definition and diagnosis. Age Ageing. 2019;48:16–31. 10.1093/ageing/afy169 PMC6322506 30312372

[10] Luo DH Wan X Liu JM Tong TJ How to estimate the sample mean and standard deviation from the sample size, median, extremes or quartiles? Chin J Evid Based Med. 2017;17:1350–6.

[11] Higgins JPT Altman DG Gotzsche PC; Cochrane Bias Methods Group. The Cochrane Collaboration’s tool for assessing risk of bias in randomised trials. BMJ. 2011;343:d5928. 10.1136/bmj.d5928 PMC3196245 22008217

[12] Khalil M Di Ciaula A Jaber N Grandolfo R Fiermonte F Portincasa P. Multidimensional assessment of sarcopenia and sarcopenic obesity in geriatric patients: creatinine/cystatin C ratio performs better than sarcopenia index. Metabolites. 2024;14:306. 10.3390/metabo14060306 PMC11205317 38921440

[13] Nascimento DDC Oliveira SDC Vieira DCL. The impact of sarcopenic obesity on inflammation, lean body mass, and muscle strength in elderly women. Int J Gen Med. 2018;11:443–9. 10.2147/IJGM.S187285 PMC6254502 30538530

[14] Yang CW Li CI Li TC. Association of sarcopenic obesity with higher serum high-sensitivity C-reactive protein levels in Chinese older males - a community-based study (Taichung Community Health Study-Elderly, TCHS-E). PLoS One. 2015;10:e0132908. 10.1371/journal.pone.0132908 PMC4503458 26177029

[15] Ribeiro JC Duarte JG Gomes GAO. Associations between inflammatory markers and muscle strength in older adults according to the presence or absence of obesity. Exp Gerontol. 2021;151:111409. 10.1016/j.exger.2021.111409 34022276

[16] Kim YM Kim S Won YJ Kim SH. Clinical manifestations and factors associated with osteosarcopenic obesity syndrome: a cross-sectional study in Koreans with obesity. Calcif Tissue Int. 2019;105:77–88. 10.1007/s00223-019-00551-y 30997575

[17] Dutra MT Avelar BP Souza VC. Relationship between sarcopenic obesity‐related phenotypes and inflammatory markers in postmenopausal women. Clin Physio Funct Imaging. 2015;37:205–10. 10.1111/cpf.12287 26373437

[18] Fantin F Giani A Manzato G. Sarcopenia, sarcopenic obesity, and arterial stiffness among older adults. Front Cardiovasc Med. 2024;11:1272854. 10.3389/fcvm.2024.1272854 PMC10885346 38404726

[19] Scott D Cumming R Naganathan V. Associations of sarcopenic obesity with the metabolic syndrome and insulin resistance over five years in older men: the concord health and ageing in men project. Exp Gerontol. 2018;108:99–105. 10.1016/j.exger.2018.04.006 29649572

[20] dos Santos EP Gadelha AB Safons MP Nóbrega OT Oliveira RJ Lima RM. Sarcopenia and sarcopenic obesity classifications and cardiometabolic risks in older women. Arch Gerontol Geriatr. 2014;59:56–61. 10.1016/j.archger.2014.03.012 24766993

[21] Lu C-W Yang K-C Chang H-H Lee L-T Chen C-Y Huang K-C. Sarcopenic obesity is closely associated with metabolic syndrome. Obes Res Clin Pract. 2013;7:e301–7. 10.1016/j.orcp.2012.02.003 24306159

[22] Liu X Hao Q Yue J. Sarcopenia, obesity and sarcopenia obesity in comparison: prevalence, metabolic profile, and key differences: results from WCHAT study. J Nutr Health Aging. 2020;24:429–37. 10.1007/s12603-020-1332-5 PMC12876396 32242211

[23] Yen C-H Chang P-S Chang Y-H Lin P-T. Identification of coenzyme Q10 and skeletal muscle protein biomarkers as potential factors to assist in the diagnosis of sarcopenia. Antioxidants (Basel). 2022;11:725. 10.3390/antiox11040725 PMC9030756 35453410

[24] Ying L Zhang Q Yang YM Zhou JY. A combination of serum biomarkers in elderly patients with sarcopenia: a cross-sectional observational study. Int J Endocrinol. 2022;2022:4026940. 10.1155/2022/4026940 PMC8885259 35237317

[25] Yee ML Hau R Taylor A. Sarcopenia in women with hip fracture: a comparison of hormonal biomarkers and their relationship to skeletal muscle mass and function. Osteoporos Sarcopenia. 2020;6:139–45. 10.1016/j.afos.2020.06.001 PMC7573494 33102808

[26] Wumaer A Maimaitiwusiman Z Xiao W. Plasma tumor necrosis factor-α is associated with sarcopenia in elderly individuals residing in agricultural and pastoral areas of Xinjiang, China. Front Med (Lausanne). 2022;9:788178. 10.3389/fmed.2022.788178 PMC9492969 36160136

[27] Aldisi D Abulmeaty MMA Alsaawi TA. Diagnostic value of inflammatory markers in elderly Arab women with sarcopenia. Diagnostics (Basel). 2022;12:2293. 10.3390/diagnostics12102293 PMC9600186 36291982

[28] He N Zhang Y Zhang Y Feng B Zheng Z Ye H. Circulating miR-29b decrease in response to sarcopenia in patients with cardiovascular risk factors in older Chinese. Front Cardiovasc Med. 2022;9:1094388. 10.3389/fcvm.2022.1094388 PMC9810340 36606278

[29] Singhal S Singh S Upadhyay AD. Serum creatinine and cystatin C-based index can be a screening biomarker for sarcopenia in older population. Eur Geriatr Med. 2019;10:625–30. 10.1007/s41999-019-00197-x 34652732

[30] Zou J-F Li S-T Wang L-P. Diagnostic value of nutritional risk index and other indices for predicting sarcopenia in the middle-aged and elderly population of China without cancer: a ROC curve analysis. Int J Gen Med. 2024;17:2527–38. 10.2147/IJGM.S457252 PMC11152168 38841128

[31] Can B Kara O Kizilarslanoglu MC. Serum markers of inflammation and oxidative stress in sarcopenia. Aging Clin Exp Res. 2017;29:745–52. 10.1007/s40520-016-0626-2 27571781

[32] He Y Ding F Yin M. High serum AST/ALT ratio and low serum INS*PA product are risk factors and can diagnose sarcopenia in middle-aged and older adults. Front Endocrinol (Lausanne). 2022;13:843610. 10.3389/fendo.2022.843610 PMC8971564 35370985

[33] Eguchi Y Toyoguchi T Inage K. Advanced glycation end products are associated with sarcopenia in older women: aging marker dynamics. J Women Aging. 2021;33:328–40. 10.1080/08952841.2019.1697161 31770087

[34] Kameda M Teruya T Yanagida M Kondoh H. Reduced uremic metabolites are prominent feature of sarcopenia, distinct from antioxidative markers for frailty. Aging (Albany NY). 2021;13:20915–34. 10.18632/aging.203498 PMC8457568 34492634

[35] Lo C-J Lin C-M Fan C-M. Plasma acylcarnitine in elderly Taiwanese: as biomarkers of possible sarcopenia and sarcopenia. BMC Geriatr. 2023;23:769. 10.1186/s12877-023-04485-x PMC10666394 37993772

[36] Coto Montes A Boga JA Bermejo Millo C. Potential early biomarkers of sarcopenia among independent older adults. Maturitas. 2017;104:117–22. 10.1016/j.maturitas.2017.08.009 28923171

[37] Liu H-C Han D-S Hsu C-C Wang J-S. Circulating MicroRNA-486 and MicroRNA-146a serve as potential biomarkers of sarcopenia in the older adults. BMC Geriatr. 2021;21:86. 10.1186/s12877-021-02040-0 PMC7847166 33516190

[38] Yu S Chen L Zhang Y. A combined diagnostic approach based on serum biomarkers for sarcopenia in older patients with hip fracture. Australas J Ageing. 2022;41:e339–47. 10.1111/ajag.13064 35438829

[39] Hirose S Nakajima T Nozawa N. Phase angle as an indicator of sarcopenia, malnutrition, and cachexia in inpatients with cardiovascular diseases. J Clin Med. 2020;9:2554. 10.3390/jcm9082554 PMC7463846 32781732

[40] Yoo JI Chung HJ Kim BG. Comparative analysis of the association between various serum vitamin D biomarkers and sarcopenia. J Clin Lab Anal. 2021;35:e23946. 10.1002/jcla.23946 PMC8418464 34350631

[41] Shin HE Won CW Kim M. Metabolomic profiles to explore biomarkers of severe sarcopenia in older men: a pilot study. Exp Gerontol. 2022;167:111924. 10.1016/j.exger.2022.111924 35963453

[42] Shibamoto A Namisaki T Suzuki J. Hemoglobin and endotoxin levels predict sarcopenia occurrence in patients with alcoholic cirrhosis. Diagnostics (Basel). 2023;13:2218. 10.3390/diagnostics13132218 PMC10340466 37443613

[43] Xu R Cui S Chen L. Circulating miRNA-1-3p as biomarker of accelerated sarcopenia in patients diagnosed with chronic heart failure. Rev Invest Clin. 2022;74:276–68. 10.24875/RIC.22000151 36328028

[44] Dalbeni A Natola LA Garbin M. Interleukin-6: a new marker of advanced-sarcopenic HCC cirrhotic patients. Cancers (Basel). 2023;15:2406. 10.3390/cancers15092406 PMC10177287 37173873

[45] Tang T Xie L Tan L Hu X Yang M. Inflammatory indexes are not associated with sarcopenia in Chinese community-dwelling older people: a cross-sectional study. BMC Geriatr. 2020;20:457. 10.1186/s12877-020-01857-5 PMC7648963 33160322

[46] Shin HE Won CW Kim M. Development of multiple biomarker panels for prediction of sarcopenia in community-dwelling older adults. Arch Gerontol Geriatr. 2023;115:105115. 10.1016/j.archger.2023.105115 37422966

[47] Yin M Zhang H Liu Q. Determination of skeletal muscle mass by aspartate aminotransferase / alanine aminotransferase ratio, insulin and FSH in Chinese women with sarcopenia. BMC Geriatr. 2022;22:893. 10.1186/s12877-022-03491-9 PMC9682777 36419004

[48] Yajima T Yajima K. Serum creatinine-to-cystatin C ratio as an indicator of sarcopenia in hemodialysis patients. Clin Nutr ESPEN. 2023;56:200–6. 10.1016/j.clnesp.2023.06.002 37344074

[49] Chen Y-Y Chiu Y-L Kao T-W Peng T-C Yang H-F Chen W-L. Cross-sectional associations among P3NP, HtrA, Hsp70, Apelin and sarcopenia in Taiwanese population. BMC Geriatr. 2021;21:192. 10.1186/s12877-021-02146-5 PMC7980650 33743591

[50] Öztürk ZA Kul S Türkbeyler IH Sayiner ZA Abiyev A. Is increased neutrophil lymphocyte ratio remarking the inflammation in sarcopenia? Exp Gerontol. 2018;110:223–9. 10.1016/j.exger.2018.06.013 29928932

[51] Morawin B Tylutka A Bielewicz F Zembron-Lacny A. Diagnostics of inflammaging in relation to sarcopenia. Front Public Health. 2023;11:1162385. 10.3389/fpubh.2023.1162385 PMC10351926 37465171

[52] Chang C-L Li Y-R Wang Z-Y. Serum retinol binding protein 4 as a potential biomarker for sarcopenia in older adults. J Gerontol A Biol Sci Med Sci. 2023;78:34–41. 10.1093/gerona/glac151 35857418

[53] Wang C Jin C Yin X Liu J Liu J. Relationship between serum bilirubin concentration and sarcopenia in patients with type 2 diabetes: a cross-sectional study. J Int Med Res. 2021;49:3000605211004226. 10.1177/03000605211004226 PMC8010833 33779353

[54] Park SJ Ji E Yoo HJ. Circulating lumican as a potential biomarker for osteosarcopenia in older adults. Bone. 2024;179:116959. 10.1016/j.bone.2023.116959 37956822

[55] Wei S Nguyen TT Zhang Y Ryu D Gariani K. Sarcopenic obesity: epidemiology, pathophysiology, cardiovascular disease, mortality, and management. Front Endocrinol (Lausanne). 2023;14:1185221. 10.3389/fendo.2023.1185221 PMC10344359 37455897

[56] Gonzalez A Simon F Achiardi O Vilos C Cabrera D Cabello-Verrugio C. The critical role of oxidative stress in sarcopenic obesity. Oxid Med Cell Longev. 2021;2021:4493817. 10.1155/2021/4493817 PMC8526202 34676021

[57] Liao T Xiong L Wang X Yang S Liang Z. Mitochondrial disorders as a mechanism for the development of obese Sarcopenia. Diabetol Metab Syndr. 2023;15:224. 10.1186/s13098-023-01192-w PMC10626707 37926816

[58] Tang X Zou Y Yang S. Impaired mitophagy contributes to pyroptosis in sarcopenic obesity zebrafish skeletal muscle. Nutrients. 2025;17:1711. 10.3390/nu17101711 PMC12114099 40431451

[59] Duan Y Tao K Fang Z Lu Y. Possible-sarcopenic screening with disturbed plasma amino acid profile in the elderly. BMC Geriatr. 2023;23:427. 10.1186/s12877-023-04137-0 PMC10337190 37438737

[60] Dalle S Rossmeislova L Koppo K. The role of inflammation in age-related sarcopenia. Front Physiol. 2017;8:1045. 10.3389/fphys.2017.01045 PMC5733049 29311975

[61] Li CW Yu K Shyh-Chang N. Pathogenesis of sarcopenia and the relationship with fat mass: descriptive review. J Cachexia Sarcopenia Muscle. 2022;13:781–94. 10.1002/jcsm.12901 PMC8977978 35106971

[62] Huang J Wen T Wei H. Pathophysiological mechanisms and emerging therapeutic strategies for muscle wasting: an integrative review. Front Physiol. 2025;16:1674892. 10.3389/fphys.2025.1674892 PMC12598294 41220691

[63] Minniti G Pescinini-Salzedas LM Minniti GADS. Organokines, sarcopenia, and metabolic repercussions: the vicious cycle and the interplay with exercise. Int J Mol Sci. 2022;23:13452. 10.3390/ijms232113452 PMC9655425 36362238

[64] Feng Z Xia J Yu J. Pathophysiological mechanisms underlying sarcopenia and sarcopenic obesity: a systematic review and meta-analysis of biomarker evidence. Int J Mol Sci. 2025;26:5113. 10.3390/ijms26115113 PMC12154750 40507924

[65] Habib SS Alkahtani S Alhussain M Aljuhani O. Sarcopenia coexisting with high adiposity exacerbates insulin resistance and dyslipidemia in Saudi adult men. Diabetes Metab Syndr Obes. 2020;13:3089–97. 10.2147/DMSO.S260382 PMC7457864 32922058

[66] Salles J Chanet A Guillet C. Vitamin D status modulates mitochondrial oxidative capacities in skeletal muscle: role in sarcopenia. Commun Biol. 2022;5:1288. 10.1038/s42003-022-04246-3 PMC9700804 36434267

[67] Lu S Cao Z-B. Interplay between vitamin D and adipose tissue: implications for adipogenesis and adipose tissue function. Nutrients. 2023;15:4832. 10.3390/nu15224832 PMC10675652 38004226

[68] Meyer F Bannert K Wiese M. Molecular mechanism contributing to malnutrition and sarcopenia in patients with liver cirrhosis. Int J Mol Sci. 2020;21:5357. 10.3390/ijms21155357 PMC7432938 32731496

[69] Romano AD Paglia A Bellanti F. Molecular aspects and treatment of iron deficiency in the elderly. Int J Mol Sci. 2020;21:3821. 10.3390/ijms21113821 PMC7313036 32481481

