# Supplementary material for: Systematic review and meta-analysis of biomarkers of sarcopenia and sarcopenic obesity
Source: Medicine (Baltimore). 2026 Sep 25;105(39):e50799. doi: 10.1097/MD.0000000000050799 (PMC13619247; doi:10.1097/MD.0000000000050799)
Supplement: Supplementary file 1 [file medi-105-e50799-s001.docx]

| **Author/year** | **Random sequence generation (selection bias)** | **Allocation concealment (selection bias)** | **Blinding of participant and personnel (performance bias)** | **Blinding of outcome assessment (detection bias)** | **Incomplete outcome data (attrition bias)** | **Selective reporting (reporting bias)** | **Other bias** |
| --- | --- | --- | --- | --- | --- | --- | --- |
| Khalil2024 | Low risk | Low risk | Unclear | Low risk | Low risk | Low risk | Unclear |
| Nascimento2018 | Low risk | Unclear | Unclear | Low risk | Low risk | Low risk | Unclear |
| Yang2015 | Low risk | Unclear | Unclear | Low risk | Low risk | Low risk | Unclear |
| Ribeiro2021 | Low risk | Unclear | Low risk | Low risk | Low risk | Low risk | Unclear |
| Kim2019 | Low risk | Unclear | Unclear | Unclear | Low risk | Low risk | Unclear |
| Dutra2015 | Low risk | Unclear | Low risk | Low risk | Low risk | Low risk | Unclear |
| Fantin2024 | Low risk | Unclear | Unclear | Unclear | Low risk | Low risk | Unclear |
| Scott2019 | Low risk | Unclear | Low risk | Low risk | Low risk | Low risk | Unclear |
| Santos2014 | Low risk | Unclear | Low risk | Low risk | Low risk | Low risk | Unclear |
| Lu2013 | Low risk | Unclear | Low risk | Low risk | Low risk | Low risk | Unclear |
| Liu2020 | Unclear | Low risk | Low risk | Low risk | Low risk | Low risk | Unclear |
| Yen2022 | Low risk | Low risk | Low risk | Low risk | Low risk | Low risk | Low risk |
| Ying2022 | Low risk | Low risk | Low risk | Low risk | Low risk | Low risk | Low risk |
| Yee2022 | Low risk | Low risk | Low risk | Low risk | Low risk | Low risk | Low risk |
| Wumaer2021 | Low risk | Low risk | Low risk | Low risk | Low risk | Low risk | Low risk |
| Aldisi 2022 | Low risk | Low risk | Low risk | Low risk | Low risk | Low risk | Low risk |
| He 2022 | Low risk | Low risk | Low risk | Low risk | Low risk | Low risk | Low risk |
| Singhal2019 | Low risk | Low risk | Low risk | Low risk | Low risk | Low risk | Low risk |
| Zou 2024 | Low risk | Low risk | Low risk | Unclear | Low risk | Unclear | Low risk |
| Can 2016 | Low risk | Low risk | Low risk | Low risk | Low risk | Low risk | Low risk |
| He 2022 | Low risk | Low risk | Low risk | Low risk | Low risk | Low risk | Low risk |
| Eguchia 2019 | Low risk | Low risk | Low risk | Low risk | Low risk | Low risk | Low risk |
| Kameda2021 | Unclear | Unclear | Unclear | Unclear | Unclear | Unclear | Unclear |
| Chi-Jen Lo2023 | Low risk | Low risk | Low risk | Low risk | Low risk | Low risk | Low risk |
| Montes2017 | Low risk | Low risk | Low risk | Low risk | Low risk | Low risk | Low risk |
| Liu 2021 | Low risk | Low risk | Low risk | Low risk | Low risk | Low risk | Low risk |
| Yu 2022 | Low risk | Low risk | Low risk | Low risk | Low risk | Low risk | Low risk |
| Hirose2020 | Low risk | Low risk | Low risk | Low risk | Low risk | Low risk | Low risk |
| Yoo2021 | Low risk | Low risk | Low risk | Low risk | Low risk | Low risk | Low risk |
| Shin2022 | Low risk | Low risk | Low risk | Low risk | Low risk | Low risk | Low risk |
| Shibamoto2023 | Low risk | Low risk | Low risk | Low risk | Low risk | Low risk | Low risk |
| Xu2022 | Low risk | Low risk | Low risk | Low risk | Low risk | Low risk | Low risk |
| Dalbeni2023 | Low risk | Low risk | Low risk | Low risk | Low risk | Low risk | Low risk |
| Tang 2020 | Low risk | Low risk | Low risk | Low risk | Low risk | Low risk | Low risk |
| Shin 2023 | Low risk | Low risk | Low risk | Low risk | Low risk | Low risk | Low risk |
| Yin 2022 | Low risk | Low risk | Low risk | Low risk | Low risk | Low risk | Low risk |
| Yajima 202 | Low risk | Low risk | Low risk | Low risk | Low risk | Low risk | Low risk |
| Chen 2021 | Low risk | Low risk | Low risk | Low risk | Low risk | Low risk | Low risk |
| Öztürk 2018 | Low risk | Low risk | Low risk | Low risk | Low risk | Low risk | Low risk |
| Morawin 2023 | Low risk | Low risk | Low risk | Low risk | Low risk | Low risk | Low risk |
| Chang 2022 |  |  |  |  |  |  |  |
| Wang 2021 |  |  |  |  |  |  |  |
| Park 2023 |  |  |  |  |  |  |  |

Table S1. Risk of Bias of Included Literatures
